# A case report of secondary ectopic pregnancy on the hepatic diaphragmatic surface originating from tubal abortion detected by the second laparoscopy after primary salpingectomy

**DOI:** 10.1097/MD.0000000000042968

**Published:** 2025-06-20

**Authors:** Ting Liang, Yang Ming, Hongying Li, Xin Du, Jing Jin, Tongfu Feng

**Affiliations:** a Department of Gynecology, Hubei Maternal and Child Health Hospital, Tongji Medical College, Huazhong University of Science and Technology, Hubei Province, China; b Department of School of Medicine, Wuhan University of Science and Technology, Wuhan, Hubei Province, China; c Department of Gynecology, Hubei Maternal and Child Health Hospital, Hubei Province, China.

**Keywords:** ectopic pregnancy, laparoscopic salpingectomy, secondary abdominal pregnancy, tubal abortion

## Abstract

**Rationale:**

Ectopic pregnancies tend to occur in the fallopian tubes. Secondary abdominal pregnancies (SAP) are much rarer. Laparoscopic salpingectomy is a commonly performed radical surgery. However, even if a patient has undergone radical surgery, we cannot take it lightly.

**Patient concerns:**

We report a relatively rare case here: an SAP on the hepatic diaphragmatic surface originating from a tubal abortion detected by a 2nd laparoscopy after primary salpingectomy. The patient was admitted to the local hospital for “ectopic pregnancy” half a month ago and underwent laparoscopic left salpingectomy and right tubal ligation 45 days later. But her values of beta-human chorionic gonadotropin (β-hCG) continued to rise and she experienced pain in the right shoulder, right subclavian, and right lower abdominal (Murphy sign) after the 1st surgery.

**Diagnoses:**

The final diagnosis was secondary ectopic pregnancy at the hepatic phrenic surface.

**Interventions:**

Half a month after the 1st operation, the patient underwent a laparoscopic examination in our hospital.

**Outcomes:**

After the 2nd operation, her β-hCG value decreased to normal and menstruation resumed.

**Lessons:**

In the face of ectopic pregnancy, we need to continue to monitor the β-hCG value. SAP may occur even if it is relatively rare. Pay attention to the collection of symptoms and signs of patients to reduce missed diagnoses. It is vital for physicians to control the timing of surgery. Sometimes, it is necessary to emphasize the importance of comprehensive exploration and rapid pathological examination during the operation.

## 1. Introduction

Ectopic pregnancy is the leading cause of maternal mortality in early pregnancy, accounting for 5% to 10% of all pregnancy-related deaths.^[1]^ The most common site of ectopic pregnancy is the fallopian tube; abdominal pregnancy occurs in only 1 in 10,000 cases, with a maternal mortality rate of 6%.^[2]^ Previously reported intraperitoneal pregnancy implantation sites include the omentum, peritoneum, uterine surface, and abdominal organs including the spleen, intestine, liver, abdominal large blood vessels, and diaphragm. Thus, treatment plans for ectopic pregnancy should be individualized according to the patient’s clinical manifestations, ultrasonic examination, and beta-human chorionic gonadotropin (β-hCG) level.

Laparoscopy is currently the gold standard for diagnosis and treatment of ectopic pregnancy, including conservative salpingostomy and radical salpingectomy.^[3]^ Persistent ectopic pregnancy (PEP) may occur after conservative salpingotomy because of residual trophoblasts.^[4]^ However, whether PEP or secondary abdominal pregnancy (SAP) is less likely in patients who have undergone radical resection remains to be seen. Ectopic pregnancy after salpingectomy is rare and is therefore easily misdiagnosed.^[5]^ Pregnant tissue might spontaneously discharge into the abdominal cavity through a breach of the original implantation site, such as tubal abortion or uterine rupture.^[6]^ Clinical manifestations range from asymptomatic to life-threatening hypovolemic shock or death. Incidence is extremely low, and no systematic clinical studies exist; therefore, early diagnosis and treatment are extraordinarily challenging. Even if a patient has undergone radical surgery, we cannot take it lightly. Therefore, we present this rare case.

## 2. Case presentation

**Figure 1.**
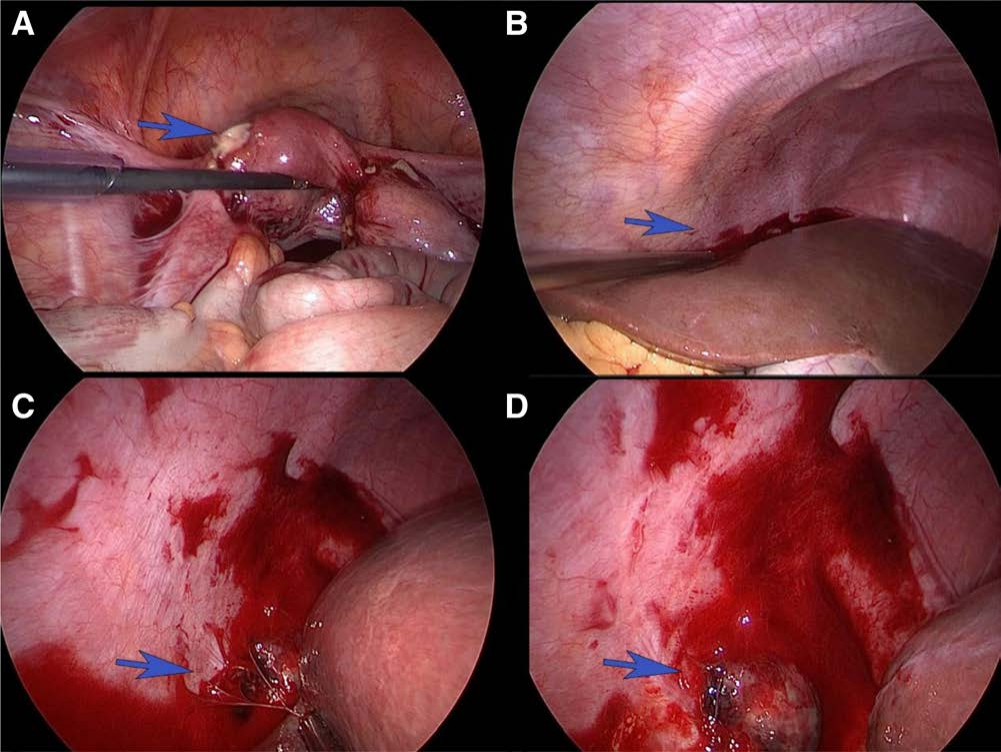
This was observed during the 2nd abdominal exploration. (A) The left fallopian tube had been resected and the right fallopian tube had been ligated. (B) Abnormal abdominal bleeding was found. (C, D) Abdominal ectopic pregnancy lesions at the junction of the right abdominal wall, diaphragm, and hepatic diaphragmatic surface.

**Figure 2.**
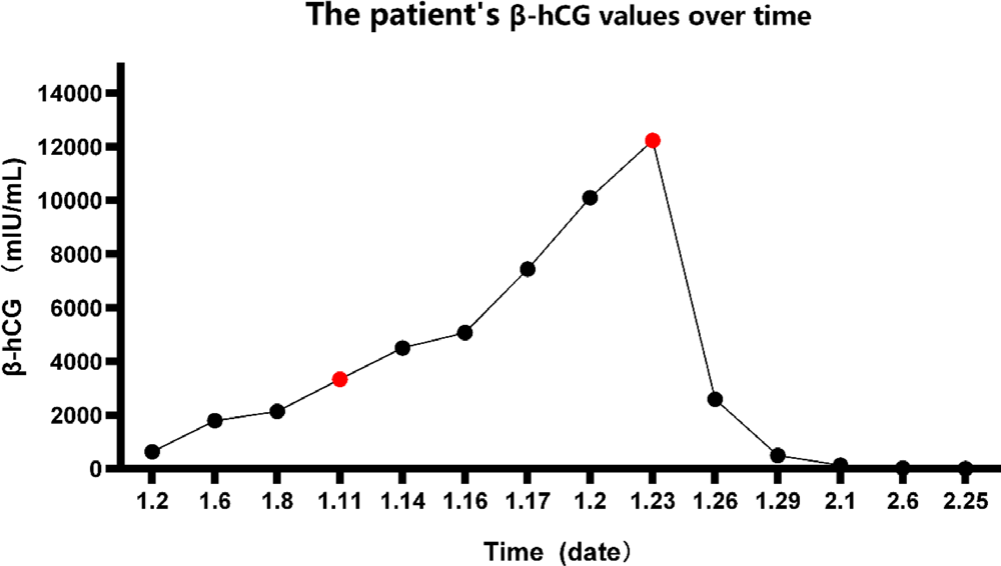
The patient’s β-hCG values over time. (Red dots indicate the time of surgery.) β-hCG = beta-human chorionic gonadotropin.

A 28-year-old woman with 3 pregnancies (2 vaginal deliveries and and 1 cesarean section) visited our secondary hospital complaining of vaginal bleeding for 6 days after left Fallopian tube resection performed due to suspected ectopic pregnancy. The patient had undergone laparoscopic left salpingectomy and right tubal ligation for ectopic pregnancy in the local hospital. Six days after hospitalization, she experienced pain in the right shoulder, right subclavian, and right lower abdomen. While in our hospital, her β-hCG value continued rising. According to records of the 1st surgery, the left fallopian ampulla was thickened by about 3.0 cm × 2.5 cm, which was purplish red on the surface and did not break. Furthermore, the umbrella’s end was covered with blood clots. Postoperative pathological examination showed that these tissues were only suspected of having villous remnants. Because we suspected the PEP, we borrowed surgical specimens from a local hospital for pathology consultation. However, we found no villous tissue. So she underwent a 2nd laparoscopic examination. A dark red and white tissue about 1.5 × 1.0 cm in size was found at the junction of the right abdominal wall, diaphragm, and hepatic diaphragmatic surface, and removed for examination (Fig. 1). Postoperative pathological diagnosis revealed many clots and villi. After the 2nd surgery, the β-hCG value gradually reduced to normal (Fig. 2). We conducted a series of follow-ups after discharge. Eventually, her menstrual cycle resumed.

## 3. Discussion

Persistent trophoblastic activity is characterized by a stable or even continuous increase in the postoperative β-hCG level. In fact, the incidence of postoperative PEP is 2.1% to 5.4%.^[4,7]^ Moreover, PEP mostly occurs in situ. Therefore, we report a rare case of hepatic diaphragmatic surface ectopic pregnancy.

Implantation of this lesion cannot currently be attributed to a medical washing procedure. Previous cases in which trophoblasts or meconium tissue were found in peritoneal washings are rare.^[8]^

Our retrospective diagnosis showed that the patient also experienced pain in the right shoulder, right subclavian, and right lower abdomen during her 1st hospitalization. This could indicate abnormal stimulation at that time. Combined with the symptoms before the 1st operation, the increasing trend of β-hCG value, and the pathological consultation results, we suspect that the patient had a tubal abortion before the operation and then the villus was implanted in the deep abdominal cavity: the junction of the right lobe of the liver, diaphragm, and abdominal wall.

Why did the patient experience symptom remission after the 1st surgery? We consider 3 reasons. First, routine abdominal irrigation at the end of the 1st surgery washed away a small amount of blood from the implantation site and the pelvic-abdominal cavity, thus reducing abdominal irritation. Second, the discomfort caused by the artificial pneumoperitoneum after the 1st laparoscopic surgery masked the original symptoms, which were not obvious. Third, this could be due to the patient’s psychology. However, the specific reasons for this phenomenon need further analysis.

Therefore, we have the following thoughts: First, even after a patient has undergone radical surgery, we must continuously monitor changes in β-hCG levels.^[9]^ Additionally, the patient’s symptoms and signs are important. Physicians should consider indirect pain and discomfort experienced by patients during the early stages of the disease. These are clinical clues that cannot be ignored.

Secondly, SAP is rare. Preoperative diagnosis is very difficult; therefore, it is usually made intraoperatively. During surgery, we need to emphasize the importance of comprehensive intraoperative exploration and, if necessary, consider the flexible use of intraoperative rapid pathologic examination techniques.

Finally, early abdominal pregnancy is difficult to detect by auxiliary examinations. If a 2nd operation is performed directly, the planted villi may not be easily detected by the naked eye, as they do not cause significant bleeding. Therefore, it is vital for physicians to control the timing of surgery.

## 4. Conclusion

In conclusion, SAP after primary laparoscopic radical surgery of tubal pregnancy is very rare. And the ectopic pregnancy on the diaphragmatic surface of the liver is even more rare. Reviewing the case can help us correct it in time and think comprehensively in future clinical practice to avoid serious consequences. We mainly hope that this case report can help us improve our understanding of the above aspects especially: When it is difficult to determine whether there are villi or other pregnant tissues in the resected tissues during the operation, it is necessary to emphasize the importance of comprehensive exploration during the operation. If required, rapid pathological examination during the operation can also be used.

## Acknowledgments

We thank the patient for participating in this case report.

## Author contributions

**Conceptualization:** Hongying Li, Xin Du.

**Data curation:** Yang Ming, Hongying Li, Xin Du.

**Formal analysis:** Ting Liang, Hongying Li, Xin Du.

**Investigation:** Ting Liang, Yang Ming, Xin Du.

**Methodology:** Yang Ming, Hongying Li, Xin Du, Jing Jin.

**Resources:** Yang Ming, Jing Jin.

**Supervision:** Xin Du, Jing Jin.

**Validation:** Xin Du, Jing Jin.

**Visualization:** Xin Du, Jing Jin, Tongfu Feng.

**Writing – original draft:** Ting Liang.

**Writing – review & editing:** Tongfu Feng.
